# Supplementary material for: Genotyping and Antimicrobial Susceptibility Profiling of Streptococcus uberis Isolated from a Clinical Bovine Mastitis Outbreak in a Dairy Farm
Source: Antibiotics (Basel). 2021 May 28;10(6):644. doi: 10.3390/antibiotics10060644 (PMC8229259; doi:10.3390/antibiotics10060644)
Supplement: Supplementary file 1 [file antibiotics-10-00644-s001.zip › antibiotics-1211642-supplementary.pdf]

antibiotics

MDPI

Table S1. Descriptive statistics of MIC values for the 14 antimicrobial agents and comparison of their distribution in the two genotypic clusters.

**Report**															
Cluster		AMC	AMP	CEZ	CEF	CPZ	CEQ	ENRO	ERY	FLL	LIN	OXA	PEN	T/S	TET
I	**Mean**	0.2308	0.2296	0.8221	0.9327	1.5577	0.4976	0.5649	0.4976	1.9808	13.2885	2.0192	0.5986	0.1409	24.8750
	**SD**	0.1222	0.2584	2.1496	2.1545	2.1251	2.1933	0.6020	2.2112	0.6101	5.3112	0.9598	2.4355	0.5500	12.3674
	**SE**	0.0170	0.0358	0.2981	0.2988	0.2947	0.3041	0.0835	0.3066	0.0846	0.7365	0.1331	0.3377	0.0763	1.7150
II	**Mean**	0.1801	0.1783	0.5368	0.4007	1.3015	0.1765	0.4706	1.0588	1.8235	14.4706	1.7721	0.1287	0.0515	23.5588
	**SD**	0.0695	0.0731	0.1965	0.2109	0.5712	0.0994	0.0830	3.8503	0.7276	4.3318	0.8152	0.0347	0.0246	11.4149
	**SE**	0.0168	0.0177	0.0477	0.0511	0.1385	0.0241	0.0201	0.9338	0.1765	1.0506	0.1977	0.0084	0.0060	2.7685
**Statistical Analysis**															
		AMC	AMP	CEZ	CEF	CPZ	CEQ	ENRO	ERY	FLL	LIN	OXA	PEN	T/S	TET
**Mann-Whitney U test**		316	384	442	231.5	420	350.5	385	427.5	376	401.5	378	377	406.5	396
**Wilcoxon W test**		469	537	595	384.5	1798	503.5	1763	580.5	529	1779.5	531	530	559.5	549
**Z**		−2.112	−0.919	0.000	−3.265	−0.370	−1.450	−0.925	−0.413	−1.249	−0.828	−1.240	−1.291	−0.536	−0.789
***p* value ***		0.034[a]	0.336	1.000	0.001 [a]	0.731	0.141	0.351	0.979	0.180	0.445	0.227	0.243	0.605	0.435
**99% CI**	*Lower confidence limit*	0.029	0.324	1.000	0.000	0.720	0.132	0.338	0.975	0.170	0.432	0.216	0.232	0.592	0.423
	Upper confidence limit	0.039	0.348	1.000	0.002	0.743	0.150	0.363	0.982	0.189	0.457	0.237	0.254	0.617	0.448

* Monte Carlo approximation two tailed. [a] Significance level ($p < 0.05$)
